# The Impact of the COVID-19 Pandemic on Physician Visits in Japan

**DOI:** 10.3389/fpubh.2021.743371

**Published:** 2021-11-01

**Authors:** Narimasa Kumagai

**Affiliations:** Faculty of Economics, Seinan Gakuin University, Fukuoka, Japan

**Keywords:** COVID-19, healthcare costs, physician visits, preschool children, state of emergency declaration

## Abstract

**Background:** Emerging from the coronavirus disease 2019 (COVID-19) scenario, fears of social distancing and contagion have led to a decline in the number of physician visits in Japan, placing severe financial strain on most hospitals and clinics. In this context, this study examined the impact of the spread of COVID-19 on the utilization of outpatient services.

**Methods:** This study used monthly data drawn from the monthly statistics report of the social insurance medical fee payment fund in Japan and estimated fixed-effects models.

**Results:** The results showed that the decline in the number of physician visits because of the first state of emergency declaration in Japan was greater than that caused by COVID-19's spread during the same period. However, there was a decline in the impact of the declaration over time. After the second state of emergency declaration, the decline in the number of physician visits caused by the spread reduced by almost half. The nationwide preschool closure under the declaration of the first state of emergency also adversely impacted the number of physician visits. The reduced healthcare per capita costs of preschool children were greater among prefectures taking specific precautions. The results showed non-negligible regional differences in physician visits of preschool children during the sample period.

**Conclusions:** The findings imply that we should not overestimate the negative impacts of the state of emergency declaration without lockdown on physician visits. To restore the number of physician visits to its pre-pandemic level, it is crucial to facilitate a smooth transition of COVID-19 patients between hospitals and an effective compensation program for hospitals with COVID-19 patients.

## Introduction

During the early phase of the COVID-19 pandemic, studies investigated the socio-demographic and economic impacts of the pandemic's onset. Findings revealed a positive correlation between population density and COVID-19 mortality or health outcome variables (1–3). Empirical evidence has indicated that the COVID-19 pandemic might have enlarged regional disparities in the healthcare access of the population worldwide.

The COVID-19 pandemic led to a decline in outpatient visits in mid-March 2020. In the United States, the decreasing trend in outpatient visits saw the lowest number of visits in mid-April 2020 (4). Boserup et al. (5) showed that the decline in the weekly emergency department visits on March 8, 2020 coincided with the World Health Organization's declaration on March 11, 2020 declaring COVID-19, which originated in Wuhan, China, as a pandemic (6).

In Japan, to minimize the impact of the country's population density on the spread of COVID-19, the country's first state of emergency was declared on April 7, covering seven prefectures—Tokyo, three surrounding prefectures (Saitama, Chiba, and Kanagawa), Osaka, Hyogo, and Fukuoka. On April 16, the areas covered by the state of emergency were expanded to all of Japan's 47 prefectures. The state of emergency declaration to stay indoors and close non-essential stores was legally non-binding. Furthermore, this declaration was not followed by a lockdown. The pandemic has given rise to social distancing and contagion fears, which has led to a decline in the number of physician visits. In Japan, the total claimed hospital charges also decreased by 7, 14, and 5% in April, May, and June 2020, respectively, over the same months in 2019 (7).

At the beginning of the outbreak, the extent of the decline in outpatient visits was not uniform across medical specialties. In addition, the impact has particularly been large for children. Studies in Norway have associated higher engagement in health-protective behaviors—such as increased hygiene or physical distancing—with households having more children (8). In Japan, parents with younger children also tend to engage in self-protective behaviors against the pandemic. Concerning parents' physician visits, a survey from April 30 to May 31, 2020 revealed that 45% of surveyed parents with symptoms refrained from physician visits due to the fear of contagion (9). The number of physical visits decreased by the nationwide preschool closure, and this scenario was further worsened by the leaves taken by nurses who had preschool children at home, owing to the school closure. According to a survey on nurses' responses to COVID-19 by the Japanese Nursing Association published on December 22, 2020, during the pandemic, nurses had reduced working days among 40% of hospitals. Most took absences due to the school closure. Furthermore, the shortage of nurses that ensued was due to a decrease in outpatient services provided.

Even during the second state of emergency declaration, the fear of contagion compelled several parents to avoid physician visits. On January 7, 2021, Japan's second state of emergency was declared, affecting Tokyo and three surrounding prefectures. On January 14, coverage was extended to an additional seven prefectures, including Aichi, Osaka, and Fukuoka. According to a report by the Japan Medical Association^1^ published on April 28, 2021, the situation worsened the financial conditions of clinics during the months from November 2020 to January 2021. Particularly, outpatient visits to pediatricians stood at ~62% in January 2021, relative to the same month the previous year. The report concluded that people refraining from physician visits diminished pediatricians' first visit fees.

Given this context, this study estimates the impact of the spread of COVID-19 on the utilization of outpatient services. Since responses were motivated by subjective rather than objective perceptions of risk (10), the study estimation function considers both the first and second states of emergency declaration. Thus, this study examines the differences in the effects of emergency declaration on physician visits. In this context, there is little evidence that the public management of infectious diseases is sensitive to changes in physician visits and disease avoidance. This study also analyzes the heterogeneous impact of COVID-19 across regions. It must be noted that the increased clinic closure owing to continual decline in the number of physician visits may increase regional disparities in healthcare access.

This study tests the following hypotheses: (1) The declaration of the state of emergency reduced new COVID-19 cases and fear of contagion and the tendency of patients to refrain from physician visits and (2) The heterogeneous impact of COVID-19 across regions and regional differences in the reduced healthcare per capita costs of preschool children. However, monthly data that show the real bed occupancy rate for COVID-19 patients are not available. Due to this data limitation, this study cannot consider the supply constraints of hospital admissions. The remainder of this paper is organized as follows. Section Methods presents the monthly trends in outpatient visits in Japan, empirical strategy, and data used in this study. Section Results presents the estimation results. Sections Discussion and Conclusion present the discussion and conclusion, respectively.

## Methods

### Monthly Trends in Outpatient Visits and the Declaration of the State of Emergency in Japan

As of Spring 2020, the expert meeting on new coronavirus disease control (EMNCDC) of the Japanese government postulated that an 80% restriction in social activity would be necessary to contain the spread of infection. Owing to the several measures that had been taken in this direction, the Japanese public fulfilled the recommendations of the EMNCDC even before the declaration of the first state of emergency in the country on April 7 (11). In this regard, Watanabe and Yabu (12) used smartphone location data and examined the effect of the declaration of the first state of emergency and the closure of schools on the stay-at-home measure. They found that the government's declaration induced people to stay indoors. They showed that the declaration of the state of emergency led to a decline in the number of people leaving their homes by 8.5% and that a 1% increase in new infections within the prefecture reduced people's outings within that prefecture by 0.027%. In the given context, it can be stated that the government's social distancing measure acted as a barrier to healthcare access.

**Figure 1:**
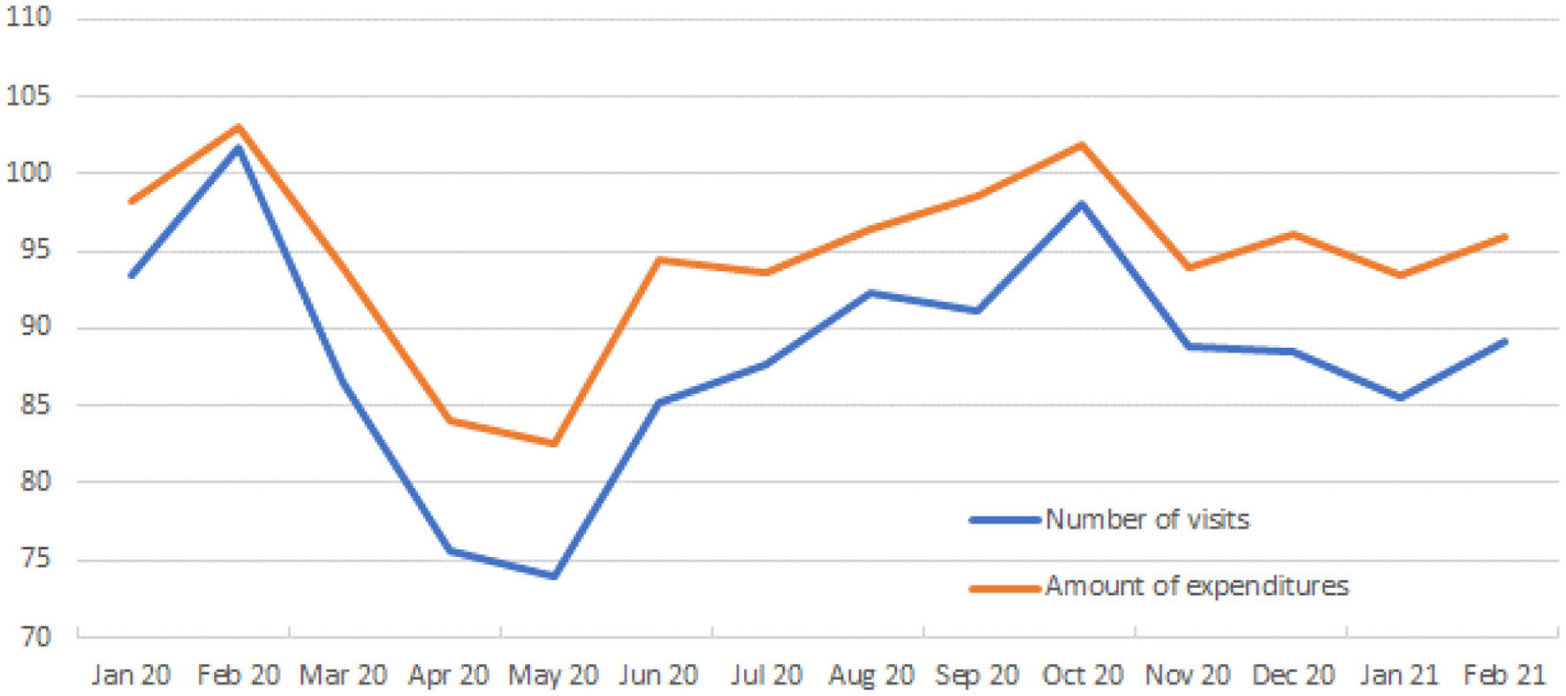
Monthly trends in outpatient visits relative to the same months in 2020–2021. This figure reports the change rate in outpatient visits relative to the same month in the previous year (January 2020–February 2021), not controlling for flu season effects. Data are from the monthly statistics report of the social insurance medical fee payment fund (*Shiharai Kikin*; https://www.ssk.or.jp/tokeijoho/geppo/index.html).

According to the monthly statistics report of the social insurance medical fee payment fund, in Japan, the pandemic led to a decline in the total number outpatient visits during the 12-month period in 2020, relative to the same period in the previous year. Figure 1 shows that the COVID-19 pandemic led to a decline in outpatient visits in March 2020. The maximum decrease in outpatient visits peaked in May 2020, when the pandemic reduced the outpatient visits by 74% relative to the same month in the previous year. The impact on outpatient visits has been declining since June 2020; however, the decline in the number of outpatient visits exerted severe financial pressures on most hospitals and clinics. The impact on outpatient visits declined in the 5 months ensuing June. Specifically, there was no difference in the outpatient expenditure between October 2020 and the same month in the previous year. However, the spread of COVID-19 led to a decline in the number of outpatient visits over the 3-month-period from November 2020 to January 2021.

**Figure 2:**
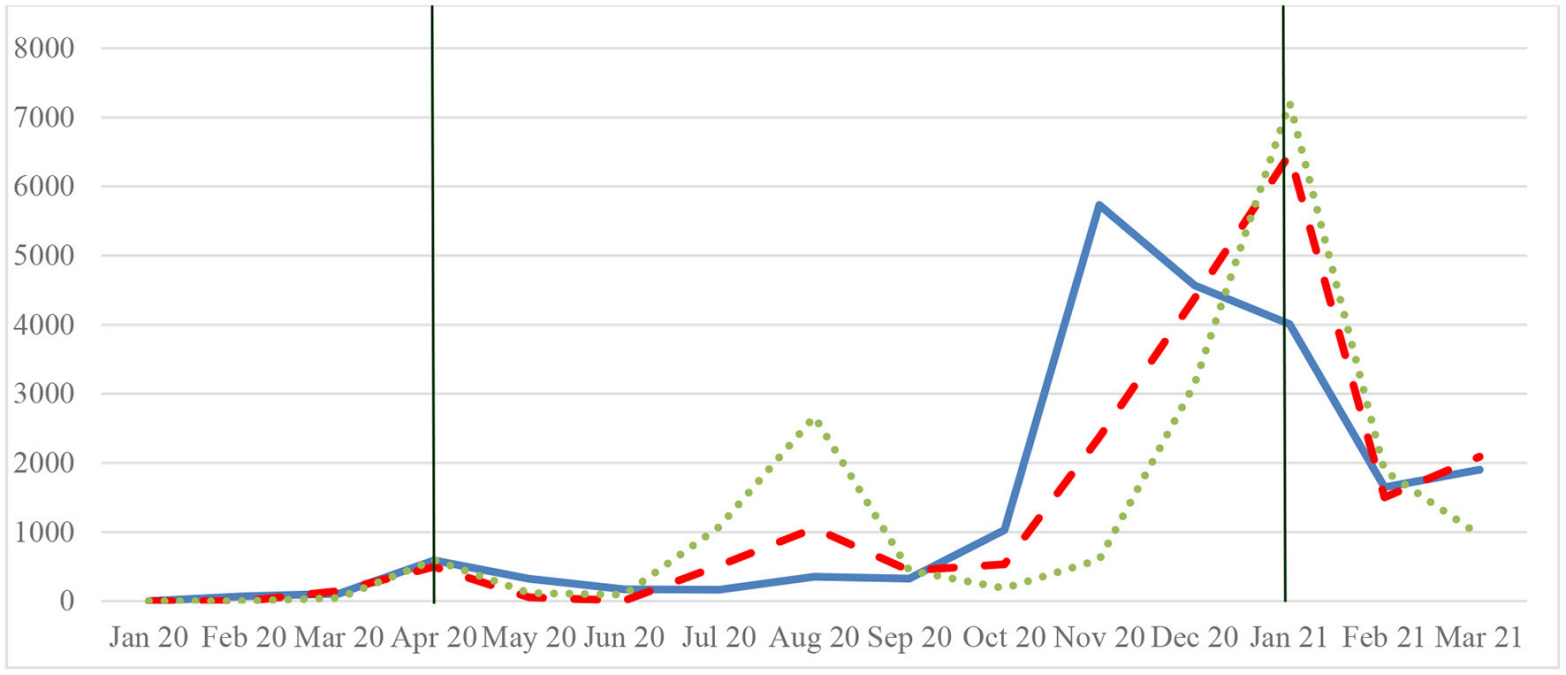
Trends in confirmed COVID-19 cases among the three prefectures. Hokkaido (blue bold line), Hyogo, and Fukuoka (dotted line), January 2020–March 2021. The daily numbers of confirmed COVID-19 cases are taken from the NHK (Nippon Hoso Kyokai); monthly data were created by the author. The green vertical line shows the declaration of the state of emergency.

The extent of the decline in the number of physician visits during the sample period may be related to the COVID-19 prevalence in the prefectures. For example, there were variations in the monthly trends in the number of confirmed cases among the three prefectures of Hokkaido, Hyogo, and Fukuoka during the sample period, while there was no difference in the total number of confirmed COVID-19 cases. There were little variations in the monthly trends among Saitama, Chiba, and Kanagawa (see, a dummy variable for the first state of emergency, the seven prefectures shown in Table 1). The confirmed cases in Hokkaido peaked in November, and this spread reduced people's outings within Hokkaido (see Figure 2). Conversely, the number of confirmed cases in Hyogo and Fukuoka peaked in January 2021. Fukuoka had two peaks during the sample period; however, this study did not observe the spread in the summer season in Hyogo. Regional differences in the fear of contagion might have influenced the number of physician visits.

**Table 1:** Monthly summary statistics by prefecture.

**Variables**	***N***	**Mean**	**SD**	**Minimum**	**Maximum**
Confirmed COVID-19 cases per 100,000 people	658	14.289	28.483	0	289.97
Outpatient visits relative to the same month in the previous year	658	0.895	0.070	0.66	1.22
Outpatient costs relative to the same month in the previous year	658	0.946	0.053	0.77	1.08
Number of inpatients relative to the same month in the previous year	658	0.970	0.075	0.76	1.25
Inpatient costs relative to the same month in the previous year	658	0.981	0.052	0.84	1.15
Active job openings-to-applicants ratio (seasonally adjusted)	658	1.264	0.247	0.72	2.17
**Preschool children**
Healthcare costs per preschool children (1,000 yen)	658	14.694	2.505	8.14	20.92
Population of preschool children, excluding infants (1,000 person)	658	101.192	105.854	22	535
Doctor's consultation rate (inpatients included)	658	1.470	0.307	0.73	2.24
Healthcare costs per physician visits (inpatients included)	658	10.133	1.134	7.45	14.47
Monthly change rate in healthcare costs per preschool children (*hcpc*)	611	−0.001	0.145	−0.47	0.47
Monthly change rate in doctor's consultation rate	611	−0.001	0.178	−0.46	0.63
Monthly change rate in *hcpc* per physician visits	611	0.009	0.101	−0.29	0.52
**Declaration of state of emergency**
Dummy variable for the first state of emergency in seven prefectures	658	0.023	0.149	0	1
Dummy variable for the specific precautions in six prefectures	658	0.018	0.134	0	1
Dummy variable for the second state of emergency in 11 prefectures	658	0.033	0.180	0	1

*Health data were collected from the monthly statistics report of social insurance medical fee payment fund (Shiharai Kikin). The active job openings-to-applicants ratio was collected from the Employment Referrals for General Workers, the Ministry of Health, and Labor and Welfare*.

*Children below 5 years, as of October 2019, were used as a proxy variable of the population of preschool children, excluding infants during the sample period. 14 months ×47 prefectures = 658*.

There was a sharp drop in the hospitalization rate of patients with asthma during the COVID-19 pandemic (13). Further, there was also a decline in the number of patients with other infectious diseases, such as influenza. According to a report by the National Institute of Infectious Diseases in Japan, the signs of seasonal influenza epidemics in Japan have still not been identified (14). With regard to COVID-19, area hospitals struggled to keep up with COVID-19 hospitalizations during the second state of emergency.

### Empirical Strategy

**Figure 3:**
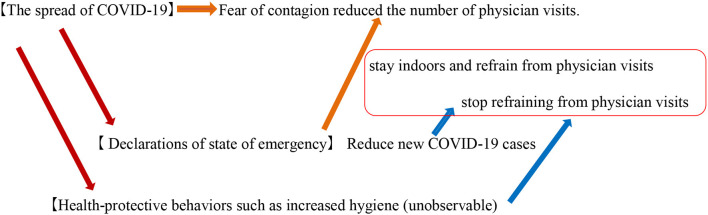
Influence of the state of emergency declaration.

The estimation function measures the direct effect of the spread of COVID-19 on physician visits and the indirect effect of the declaration of the state of emergency in reducing new COVID-19 cases by reducing the fear of contagion and practice of refraining from physician visits (see Figure 3). The other unobservable effect of health-protective behaviors, such as increased hygiene, might have a positive effect on physician visits. The core econometric specification of this function is as follows:


(A) $$\begin{array}{l} Y_{i,t}=C+Z_{i,t}\beta^{\prime }+D_{i,t}\gamma^{\prime }+(Z_{i,t}\times D_{i,t})\delta^{\prime }+X_{i,t}\theta^{\prime }+\tau W_{i,t}\\ \;+\mu J_{i,t}+v_{i,t}+\varepsilon_{i,t} \end{array}$$

where *Y*_*i, t*_ is a dependent variable, such as the number of outpatients relative to the same month in the previous year. The subscripts *i* and *t* indexes the prefecture and time periods, respectively. ***Z*** is a vector of confirmed COVID-19 cases per 100,000 people (current and lagged), and ***D*** is a vector of dummy variables for the first or second states of emergency or specific precautions. *W* is a dummy variable for the winter season, *J* is the active job openings-to-applicants ratio (seasonally adjusted), and *C* is a constant term. The vector ***X*** includes the reciprocal variable of the change rate in outpatient or inpatient costs and the number of inpatients or outpatients (current and lagged). To avoid the endogeneity problem in which physician visits and healthcare costs are determined simultaneously, the study uses lagged reciprocal variables. β′, γ′, δ′, θ′, τ, and μ are the coefficients to be estimated. The error term is *v*_*i, t*_ + ε_*i, t*_; *v*_*i, t*_ is the unit-specific constant error term; all the unobserved time-invariant components, ε_*i, t*_, are assumed to be independent of covariates in Equation (A).

The fixed-effects model considers two aspects—the positive and negative effects of the declaration of the state of emergency on physician visits. Under the declaration of the state of emergency, some people tended to stay indoors and refrain from physician visits. Conversely, people have less fear of contagion when there is a decline in the number of confirmed COVID-19 cases. To capture the interaction effect of the confirmed COVID-19 cases and the declaration of the state of emergency on physician visits, the study used the interaction term of those variables (***Z***_*i, t*_**×*D***_*i, t*_**)**. When there is a decline in the fear of contagion and an increase in the number of outings, relative to staying indoors, the interaction term exerts a positive effect on physician visits (a square box in Figure 3). In this regard, Boone and Ladreit (15) showed that an aggregate measure of the level of stringency of non-pharmaceutical interventions was associated with greater reductions in mobility in advanced member countries of the Organization for Economic Co-operation and Development (OECD) during the first wave. The aggregate measure, an index similar to that of the Oxford Stringency Index, includes school closure, workplace closure, the cancelation of public events, the restrictions on gatherings and internal movements, the closure of public transport, stay-at-home requirements, and international travel controls. Based on their results, it is considered that the nationwide preschool closure, under the declaration of the state of emergency, adversely influences the number of physician visits. This impact was heterogeneous across regions owing to the degree of reduction in mobility. In this context, this study investigates regional differences in the reduced healthcare per capita costs of preschool children.

### Data

This study uses monthly data from January 2020 to February 2021 and estimates the fixed-effects models. Data were drawn from the monthly statistics report of the social insurance medical fee payment fund (*Shiharai Kikin* in Japanese) in Japan. During the sample period, relative to the same period in the previous year, there was an almost 10 and 5% decline in the number of outpatients and their healthcare costs, respectively (Table 1). The minimum value of the monthly change rate in healthcare costs per preschooler and doctor's consultation rate for preschool children were −0.47, and −0.46, respectively. With regard to new COVID-19 cases, the mean of the monthly confirmed COVID-19 cases per 100,000 people during the sample period was 14.29, with a standard deviation of 28.48. Therefore, COVID-19 cases per 100,000 people exhibited a fat-tailed distribution because the upper interval of one standard deviation from the mean was (SD = 42.78, 289.97).

In the given context, it must be noted that the COVID-19 pandemic in Japan led to a deterioration of the employment situation amid the social distancing and temporary business closures. The ratio of active job openings-to-applicants was 1.20 (seasonally adjusted value) in May 2020—a year-on-year decrease of 0.12% points. This was the country's second-largest monthly decline in history, following the 0.20% point fall in February 1974. The ratio of active job openings-to-applicants indicates the number of jobs from companies per active job seeker. For the first state of emergency, the seven prefectures of Saitama, Chiba, Tokyo, Kanagawa, Osaka, Hyogo, and Fukuoka took a value of 1 as a dummy variable, and 0 otherwise. For the specific precautions in the prefectures, the six prefectures of Hokkaido, Ibaragi, Ishikawa, Gifu, Aichi, and Kyoto took a value of 1 as a dummy variable, and 0 otherwise. For the second state of emergency, the 11 prefectures of Tochigi, Saitama, Chiba, Tokyo, Kanagawa, Gifu, Aichi, Kyoto, Osaka, Hyogo, and Fukuoka took a value of 1 as a dummy variable, and 0 otherwise.

## Results

This study used the Hausman's specification test to compare the results of the random-effects model with that of the fixed-effects model. The test supported the initial hypothesis that the prefectural-level effects are adequately modeled by a fixed-effects model at the 1% level (not shown). Table 2 shows the estimation results for the number of outpatients during the periods April–May 2020 and January–March 2021, relative to the same months in the previous years. Equation (2) includes the active job openings-to-applicants ratio as a variable of the business cycle, which was not significant at the 5% level. The major findings from Equation (1) are summarized as follows: First, when there were 110 new confirmed cases per 100,000 people in the past month (Stage 4: explosive spread of infection), the spread of COVID-19 had reduced 10% of the number of physician visits relative to that in the previous year. There is comparability in the magnitude of the following two impacts on the number of physician visits: the first state of emergency declaration in the seven prefectures and the 82 new confirmed cases per 100,000 people in a month (Stage 3: rapid increases in infected people). Second, after the second state of emergency declaration, the decline in the number of physician visits caused by the spread reduced by almost half (−0.555 = 0.000539/−0.000971). However, the second declaration had a negative impact on the number of inpatients.

**Table 2:** Number of outpatients.

**Variables**	**(1)**	**(2)**
**Dependent variable: number of outpatients relative to the same month in the previous year**		
Confirmed COVID-19 cases per 100,000 people	−0.000971^***^	−0.00102^***^
	(0.000115)	(0.000129)
Confirmed COVID-19 cases per 100,000 people (lagged)	−0.000542^***^	−0.000574^***^
	(0.000118)	(0.000123)
Dummy variable for the first state of emergency (April–May 2020)	−0.0803^***^	−0.0787^***^
	(0.0195)	(0.0196)
Dummy variable for prefectures taking specific precautions (April–May 2020)	−0.0614^***^	−0.0599^***^
	(0.0207)	(0.0208)
Dummy variable for the second state of emergency (January–March 2021)	0.00315	0.00360
	(0.0235)	(0.0235)
Confirmed COVID-19 cases per 100,000 people × the first state of emergency	−0.000896	−0.000955
	(0.00180)	(0.00180)
Confirmed COVID-19 cases per 100,000 people × the specific precautions	0.000196	0.000180
	(0.00241)	(0.00241)
Confirmed COVID-19 cases per 100,000 people × the second state of emergency	0.000539^***^	0.000569^***^
	(0.000189)	(0.000192)
1/change rate in outpatient costs (lagged)^#^	−0.289^***^	−0.308^***^
	(0.0451)	(0.0496)
Number of inpatients^#^	0.630^***^	0.621^***^
	(0.0431)	(0.0445)
Number of inpatients (lagged)^#^	−0.232^***^	−0.226^***^
	(0.0494)	(0.0498)
Dummy variable for winter season	0.0280^***^	0.0328^***^
(1 = December, January, and February, and 0 otherwise)	(0.00539)	(0.00763)
Active job openings-to-applicants ratio (Seasonally adjusted, workplace)		−0.0179
		(0.0201)
Constant term	0.828^***^	0.874^***^
	(0.0872)	(0.101)
*N*	611	611
R-squared	0.585	0.585

*Standard errors are in the parenthesis*.

*** *p < 0.01*.

# *Relative to the same month in the previous year. 13 months × 47 prefectures = 611*.

The interaction term of the second state of emergency was positive and significant, whereas the other coefficients of the interaction terms were not significant at the 5% level. The positive sign implies that a decline in the fear of contagion among people reduced their tendency to stay at home during the second state of emergency^2^. The total coefficient of the second state of emergency was positive, while it was −0.08 in the first state of emergency. This indicates a decline in the impact of the declaration over time.

The results also show a significant change in the active job openings-to-applicants ratio in Equation (4). A 0.2% decrease in the active job openings-to-applicants ratio increased the number of inpatients by 2.1%, relative to the same month in the previous year. Concerning inpatients, the number of inpatients during the winter season was 5% greater than that in the other seasons, though the coefficient was 2.8% for the number of outpatients (see Equation 1). When there were 110 new confirmed cases per 100,000 people (Stage 4), the spread of COVID-19 had increased by 3% relative to that in the previous year.

The mean lengths of stay of the confirmed and suspected COVID-19 patients between April and June 2020 were 16.9 and 14.1 days, respectively (7). However, mild or non-severe COVID-19 patients, who did not have fever, did not require a longer duration of hospitalization during the second state of emergency, relative to the severe patients. The estimation result indicated that the rate of decline in the number of inpatients during the second state of emergency was almost the same as that in Stage 4 when there were 135 new confirmed cases per 100,000 people (Table 3). The estimated coefficient of the dummy variable for the second state of emergency (-0.0421) was equivalent to 0.000313 × 135 confirmed cases per 100,000 people. This implies that the average impact of the second state of emergency on the reduction in hospital admissions was the same in the specific prefecture where infection spread explosively under the first state of emergency declaration. The same pattern was not observed during the first state of emergency.

**Table 3:** Number of inpatients.

**Variables**	**(3)**	**(4)**
**Dependent variable: number of inpatients relative to the same month in the previous year**		
Confirmed COVID-19 cases per 100,000 people	0.000636^***^	0.000313^**^
	(0.000111)	(0.000124)
Confirmed COVID-19 cases per 100,000 people (lagged)	0.000852^***^	0.000667^***^
	(0.000107)	(0.000110)
Dummy variable for the first state of emergency (April–May 2020)	−0.0172	−0.00974
	(0.0184)	(0.0180)
Dummy variable for prefectures taking specific precautions (April–May 2020)	−0.0325^*^	−0.0240
	(0.0194)	(0.0190)
Dummy variable for the second state of emergency (January–March 2021)	−0.0464^**^	−0.0421^**^
	(0.0219)	(0.0214)
Confirmed COVID-19 cases per 100,000 people × the first state of emergency	−0.00293^*^	−0.00305^*^
	(0.00167)	(0.00163)
Confirmed COVID-19 cases per 100,000 people × the specific precautions	−0.000958	−0.000947
	(0.00224)	(0.00219)
Confirmed COVID-19 cases per 100,000 people × the second state of emergency	−0.000326^*^	−0.000137
	(0.000178)	(0.000177)
1/change rate in inpatient costs (lagged)^#^	0.0259	−0.0252
	(0.0433)	(0.0434)
Number of outpatients^#^	0.455^***^	0.422^***^
	(0.0338)	(0.0336)
Number of outpatients (lagged)^#^	0.250^***^	0.348^***^
	(0.0355)	(0.0393)
Dummy variable for winter season	0.0234^***^	0.0507^***^
(1 = December, January, and February, and 0 otherwise)	(0.00496)	(0.00706)
Active job openings-to-applicants ratio (Seasonally adjusted, workplace)		−0.105^***^
		(0.0197)
Constant term	0.287^***^	0.408^***^
	(0.0672)	(0.0695)
*N*	611	611
R-squared	0.672	0.688

*Standard errors are in the parenthesis*.

*** *p < 0.01*,

** *p < 0.05, and*

* *p < 0.1*.

# *Relative to the same month in the previous year. 3 months × 47 prefectures = 611*.

Concerning the preschool children, since the sole data of the preschoolers' total healthcare costs can be used for estimation, we obtained a series of healthcare costs per preschooler (1,000 yen) by dividing the total healthcare costs by the population of preschool children. Healthcare costs per preschool child include the costs of outpatients and inpatients. Owing to limited available information, children below 5 years (as of October 2019) were used as a proxy variable for the population of preschool children, excluding infants, during the sample period.

The active job openings-to-applicants ratio positively influenced the healthcare costs of preschool children (Table 4). Related to a good/bad economy, the results showed non-negligible regional differences in the physician visits of preschool children during the sample period. Among the six prefectures taking specific precautions, the reduced healthcare costs per preschooler were greater than those of the seven prefectures where the first state of emergency was declared. Since the ratio of the estimated coefficients was 1.28 (=2.471/1.926), the results showed a non-negligible regional difference in the effect of the declaration on the reduced healthcare costs per preschooler. However, there was a relatively small regional difference in the doctor's consultation rate (1.05 = 0.283/0.269, Equation 8 in Table 5), suggesting a regional difference in preschool children's healthcare costs per visit. However, the reason for this could not be determined because of the lack of patient diagnosis information.

**Table 4:** Healthcare costs of preschool children.

**Variables**	**(5)**	**(6)**
**Dependent variable: healthcare costs per preschool children**		
Confirmed COVID-19 cases per 100,000 people	−0.0166^***^	−0.00722^*^
	(0.00352)	(0.00388)
Confirmed COVID-19 cases per 100,000 people (lagged)	−0.0120^***^	−0.00590
	(0.00348)	(0.00359)
Dummy variable for the first state of emergency (April–May 2020)	−1.501^**^	−1.926^***^
	(0.591)	(0.583)
Dummy variable for prefectures taking specific precautions (April–May 2020)	−2.050^***^	−2.471^***^
	(0.625)	(0.616)
Dummy variable for the second state of emergency (January–March 2021)	0.403	0.228
	(0.717)	(0.701)
Confirmed COVID-19 cases per 100,000 people × the first state of emergency	−0.211^***^	−0.209^***^
	(0.0547)	(0.0534)
Confirmed COVID-19 cases per 100,000 people × the specific precautions	−0.0226	−0.0264
	(0.0735)	(0.0719)
Confirmed COVID-19 cases per 100,000 people × the second state of emergency	0.00174	−0.00390
	(0.00580)	(0.00577)
1/change rate in outpatient costs (lagged)^#^	−11.48^***^	−9.080^***^
	(0.663)	(0.795)
Dummy variable for winter season	2.290^***^	1.486^***^
(1 = December, January, and February, and 0 otherwise)	(0.150)	(0.213)
Active job openings-to-applicants ratio (Seasonally adjusted, workplace)		3.127^***^
		(0.599)
Constant term	27.20^***^	20.70^***^
	(0.747)	(1.444)
*N*	611	611
R-squared	0.578	0.598

*Standard errors are in the parenthesis*.

*** *p < 0.01*,

** *p < 0.05, and*

* *p < 0.1*.

# *Relative to the same month in the previous year. 13 months × 47 prefectures = 611*.

**Table 5:** Doctor's consultation rate for preschool children.

**Variables**	**(7)**	**(8)**
**Dependent variable: doctor's consultation rate for preschool children (inpatients included)**		
Confirmed COVID-19 cases per 100,000 people	−0.00313^***^	−0.00150^***^
	(0.000420)	(0.000445)
Confirmed COVID-19 cases per 100,000 people (lagged)	−0.00227^***^	−0.00117^***^
	(0.000415)	(0.000415)
Dummy variable for the first state of emergency (April–May 2020)	−0.201^***^	−0.269^***^
	(0.0704)	(0.0672)
Dummy variable for prefectures taking specific precautions (April–May 2020)	−0.213^***^	−0.283^***^
	(0.0747)	(0.0712)
Dummy variable for the second state of emergency (January–March 2021)	0.0811	0.0538
	(0.0856)	(0.0811)
Confirmed COVID-19 cases per 100,000 people × the first state of emergency	−0.0201^***^	−0.0199^***^
	(0.00653)	(0.00618)
Confirmed COVID-19 cases per 100,000 people × the specific precautions	−0.00301	−0.00331
	(0.00879)	(0.00832)
Confirmed COVID-19 cases per 100,000 people × the second state of emergency	0.000294	−0.000651
	(0.000692)	(0.000665)
1/change rate in outpatient costs (lagged)^#^	−2.137^***^	−1.572^***^
	(0.113)	(0.127)
Dummy variable for winter season	0.300^***^	0.158^***^
(1 = December, January, and February, and 0 otherwise)	(0.0179)	(0.0243)
Active job openings-to-applicants ratio (Seasonally adjusted, workplace)		0.547^***^
		(0.0674)
Constant term	3.708^***^	2.443^***^
	(0.120)	(0.193)
*N*	611	611
R-squared	0.600	0.643

*Standard errors are in the parenthesis*.

*** *p < 0.01*.

# *Relative to the same month in the previous year. 13 months × 47 prefectures = 611*.

Frequent handwashing after outdoor activities and before touching the mouth/nose area reduced the risk of infection by 97.9 and 69.7%, respectively (16). According to a survey by Watanabe and Yoshikawa (17), the number of children with infectious diseases (e.g., influenza or pneumonia) has reduced by half. Handwashing habits contributed toward reducing children's morbidity in Japan; however, the interaction terms of the first state of emergency in Equations (6, 8) were negative and significant at the 1% level. This indicates that the staying-at-home effect led parents to refrain from physician visits, which can be attributed to their voluntary self-protection behavior in response to the epidemic (10).

## Discussion

The decline in the number of physician visits owing to the first state of emergency declaration was greater than that caused by the spread during the same period. Conversely, the second state of emergency declaration did not have a statistically significant impact on the number of physician visits. The declaration's impact declined over time, and the staying-at-home effect did not persist. Given this finding, we should not overestimate the negative impacts of the state of emergency declaration on physician visits. Conversely, in the current and past months, the number of inpatients exerted a net positive impact on the number of physician visits, indicating that the number of physician visits will return to its pre-pandemic level early in the areas reporting a smooth transition of COVID-19 patients between hospitals.

Incentive and payment systems for private healthcare providers contribute to the quality and performance of health service delivery and play key roles in pandemic preparedness (18). The effect of these systems is evident in countries where the private sector has played a significant role in the health service delivery. However, Shin et al. (7) pointed out that the compensation program focusing on patients with severe COVID-19 did not contribute effectively toward improving the Japanese healthcare system. The study used 2,739,878 inpatient and 53,479,658 outpatient cases from 195 hospitals and showed that an increase in payments for severe COVID-19 patients did not compensate for the decline in the income of hospitals with COVID-19 patients. The emergency comprehensive support grant (ECSG) per COVID-19 patient to medical institutions has not been proportional to the prefectural proportion of the cumulative COVID-19 cases. For example, according to Ito (19), Tokyo received 16.4% of the total ECSG for 26% confirmed COVID-19 cases. The support grants to medical institutions should be proportionally distributed according to COVID-19 patients' severity or density.

During the state of emergency period, the regional differences in physician visits can be attributed to the lack of compensation programs for hospitals with COVID-19 patients. Indeed, the decline in the number of physician visits has a negative effect for medical institutions from a financial perspective. However, this may also contribute to avoiding unnecessary health care use, especially in children. Since parents who have children might update their information on infections based on the number of new infections, they may have refrained from seeing a doctor for fear of infection during the second state of emergency. Thus, children who can receive medical expense subsidies that local governments generally provide might reduce unnecessary physician visits during the pandemic. This study, however, cannot explore the impact of COVID-19 on behavioral change in health care use, which should be examined by future studies.

## Conclusion

Social distancing and the fear of contagion led to a decline in the number of physician visits in Japan. In this context, this study estimated the impact of the spread of COVID-19 on the utilization of outpatient services using monthly data drawn from the monthly statistics report of the social insurance medical fee payment fund in Japan. The results showed that the decline in the number of physician visits owing to the first state of emergency declaration was greater than that caused by the spread of COVID-19 during the same period; however, the declaration's impact declined over time. The nationwide preschool closure during the first state of emergency also adversely affected the number of physician visits. The results showed the non-negligible regional differences in the physician visits of preschool children during the sample period. Hence, we should not overestimate the negative impacts of the state of emergency declaration without lockdown on physician visits. Information on the spread of infection is more important for parents that have children than the state of emergency declaration.

As stated earlier, there has been a lack of effectiveness in the compensation program for hospitals with severe COVID-19 patients. Given this, a smooth transition of COVID-19 patients between hospitals and an effective compensation program for hospitals with COVID-19 patients would play crucial roles in restoring the number of physician visits to the pre-pandemic level. Future studies should also be conducted to determine the reason for the regional difference in the healthcare costs per visit during the COVID-19.

## Data Availability Statement

Publicly available datasets were analyzed in this study. This data can be found here: the monthly statistics report of the Social Insurance Medical Fee Payment Fund (https://www.ssk.or.jp/tokeijoho/geppo/index.html).

## Author Contributions

NK was responsible for the conceptualization of the study, the formal study analysis, the writing of the original draft, the review of the tasks throughout the study, and he was accountable for the content of the work.

## Funding

This study was supported by the Japanese Ministry of Education, Culture, Sports, Science, and Technology (Grant No. 20K01739). The funder had no role in the design of the study; the collection, analysis, and interpretation of the data; and the writing of the manuscript.

## Conflict of Interest

The author declares that the research was conducted in the absence of any commercial or financial relationships that could be construed as a potential conflict of interest. The handling editor declared a past co-authorship with the author.

## Publisher's Note

All claims expressed in this article are solely those of the authors and do not necessarily represent those of their affiliated organizations, or those of the publisher, the editors and the reviewers. Any product that may be evaluated in this article, or claim that may be made by its manufacturer, is not guaranteed or endorsed by the publisher.

## Footnotes

^1^The report in Japanese is available at: https://www.med.or.jp/dl-med/teireikaiken/20210428_3.pdf.

^2^The daily liquidity population data collected by the Agoop Corp. show that there was no decline in population within 500 m of the major terminal station during the second state of emergency, relative to those of the first state of emergency (https://corporate-web.agoop.net/pdf/covid-19/agoop_analysis_coronavirus.pdf).
